# ChromoCheck: Predicting Postnatal Chromosomal Trisomy Cases Using a Support Vector Machine Learning Model

**DOI:** 10.3390/genes16060695

**Published:** 2025-06-08

**Authors:** Nabras Al-Mahrami, Nuha Al Jabri, Amal A. W. Sallam, Najwa Al Jahdhami, Fahad Zadjali

**Affiliations:** 1 Medical Laboratory Sciences Program, Health Sciences, Oman College of Health Sciences, P.O. Box 3720, Muscat 112, Oman; nabras.almahrami@ochs.edu.om (N.A.-M.); nuha.aljabri@ochs.edu.om (N.A.J.); 2 Department of Clinical and Chemical Pathology, Research Institute of Ophthalmology, Giza 12511, Egypt; asallam.bsc.ihs@gmail.com; 3 Oman College of Health Sciences, P.O. Box 3720, Muscat 112, Oman; fahad.zadjali@moh.gov.om

**Keywords:** chromosome, trisomy, machine learning, support vector machine

## Abstract

Introduction: Chromosomal study via karyotype is one of the historical gold-standard procedures used to provide a clearer view of chromosomal trisomy abnormalities. It has been used to correlate several phenotypic manifestations that require immediate medical intervention. However, the laboratory procedure persisted with various drawbacks. The recent machine learning model shed light on prediction capabilities in the medical field. In this study, we aimed to use a support vector machine model for predicting postnatal chromosomal trisomy cases. Methods: A dataset of 946 neonatal records from the Royal Hospital, Muscat, Oman, covering the period from 2013 to 2023, has been used in this model. The model is based on features such as thyroxine hormone levels and thyroid-stimulating hormone levels. With different R packages, we used a support vector machine model with leave-one-out cross-validation and ten iterations to test three kernel functions: linear, radial, and polynomial. Results: Among the obtained kernel performances, the linear kernel has optimal classification performance. The training accuracy was 81%, and the testing accuracy was 82%. Sensitivity ranged from 97 to 98%, and specificity ranged from 79 to 80%. The area under the curve in relation to the training dataset came to 0.89, and it came to 0.90 for the test dataset. We deployed the trained models in a website tool called ChromoCheck. Conclusions: Our study is an example of how machine learning can be instrumental in augmenting conventional methods of cytogenetics diagnosis and decision-making in a clinical setup.

## 1. Introduction

The discovery of 46 chromosomes in human cells in the late 1950s has rapidly correlated with significant breakthroughs, such as the association of chromosomal aneuploidies or structural rearrangement with phenotype manifestation [1,2]. Since then, chromosomal studies have become an essential means for the identification of multiple chromosomal syndromes. A normal human cell has 23 chromosomal pairs, including 22 pairs of autosomes and 1 pair of sex chromosomes, either XX or XY. Changing chromosomal numbers or structures can lead to certain diseases, conditions, or cancers [3,4,5]. Trisomy cases, such as Down syndrome (trisomy 21) [6], Patau syndrome (trisomy 13) [7], and Edward syndrome (trisomy 18) [7], are the most prevalent autosomal syndromes. Typically, clinical features examinations of trisomy cases can provide a differential diagnosis. However, due to the diverse range of severity for each condition and the overlapping clinical features, the karyotype test has become the gold standard by which to define the extra chromosomal copy. This test is prepared from mitotic cells, where the cell cycle is arrested at the metaphases or prometaphases stage [8]. It is common practice to use a series of chemical treatments, such as mitotic inhibitors, hypotonic treatment, and fixatives, to harvest the cells. Next, the harvested cells are mainly processed into slide preparation and banding steps to present well-distinguished chromosomes. The manual preparation of chromosomal studies is labor-intensive and time-consuming. More advanced techniques for chromosomal aneuploidy detection, such as quantitative fluorescent polymerase chain reaction (QF-PCR), have been rapidly adopted in hospital settings [9]. Polymerase chain reaction utilizes polymorphic short tandem repeat (STR) specific markers to allocate targeted chromosomes, facilitating quantification-specific targeted chromosome sequences. This method has emerged as a rapid process by which to deliver results within 24 to 48 h, which is significantly faster than conventional karyotype tests. However, it is important to note that cases such as chromosomal trisomies are correlated to a variety of birth abnormalities such as cardiac malformation and dysmorphological features. The early detection is critical for the proper treatment and management of the disorder. While karyotyping and QF-PCR are still in use for confirming chromosomal abnormalities, they are time-consuming and require specialized laboratory infrastructure. Considering the increasing demand for rapid diagnostics, artificial intelligence (AI) provides new opportunities for developing predictive screening models.

Currently, with the increasing availability of big data, AI has successfully been used in different aspects of the medical field [10]. The machine learning (ML) approach is primary application of AI in this domain. It is focused on the creation and deployment of computer algorithms that process inputs, optimize error rates, and predict outputs [11]. These algorithms are effective enough to cope with enormous datasets that are characterized by incoherence, noise, and high dimensionality, with few indications of the underlying data distribution [11]. Currently, there are two main types of machine learning algorithms: unsupervised and supervised. The unsupervised approaches are mostly used for clustering and feature extraction and include applications like k-means clustering, principal components analysis, and self-organizing maps [12]. The supervised approaches include applications like support vector machines (SVMs) [13], random forest (RF) [14], and k-nearest neighbor (kNN) [15] and are commonly employed for classification or numeric value prediction [12]. The major distinction between unsupervised and supervised approaches is the labels of the inputs. More precisely, the unsupervised approach uses unlabeled inputs, while the supervised approach does not [16]. Somewhere in between unsupervised and supervised methods are semi-supervised learning approaches that rely on the partial labeling of input data during the training/learning procedure [12]. Currently, there have been few attempts to use ML to predict trisomy phenotypes. In 2023, Baldo et al. [17] investigated the use of random forest and gradient boosting machine models in 106 DS cases with 109 associated features. Interestingly, the model showed results with a low error (MSE < 0.12) and an acceptable R2 (0.70 and 0.93). In 2018, the SVM model was utilized for prenatal risk assessment based on maternal clinical features. The model showed an area under the curve of 0.95 and a detection rate of 61%, with a 1% false-positive rate on the test dataset [18]. While a few existing models are available, their predictive performance has room for improvement. According to several studies, the complementary predictive performance of each tool depends on the selected distinct features, the population [19], and the applied machine learning algorithms [13,14]. The main aim of this study is to test the ability of the SVM machine learning model to predict trisomy cases.

## 2. Methods

### 2.1. The Ethical Statement

All experiments were performed according to relevant guidelines and regulations under ethical approval MoH/CSR/23/26817 from the Royal Hospital, Ministry of Health (Muscat, Oman).

### 2.2. Data Collection

In this study, datasets were retrieved from the recording system at the National Genetic Center, Royal Hospital (Muscat, Oman), from 2013 to 2023. Patient information was deidentified for research purposes. As a routine procedure in the Royal Hospital, all the newborns were evaluated by different laboratory tests. In this study, we limited the associated features to the following: chromosomal karyotype result; thyroxine (T4) hormone; thyroid-stimulating hormone (TSH); and age and gender information. These features were the primary ones selected because they were routinely applied to any newborn with a suspected trisomy condition. The inclusion criteria for the study included the following: (i) newborns aged 0 to 12 months at the time of examinations; (ii) cases presenting with clinical phenotypic traits expressive of trisomy condition, such as (congenital defects, developmental delays or distinctive facial features); and (iii) cases that had been tested for karyotype and T4 and TSH hormones. Conversely, the exclusion criteria were as follows: (i) cases with a confirmed diagnosis of infection or inflammatory conditions; (ii) cases older than 1 year old at the time of karyotype and T4 and TSH hormones evaluations; (iii) cases for which we could not define the time of the evaluations; and (v) newborns with mosaicism in the karyotype results. The retrieved raw data came to *n* = 946 cases that met the inclusion criteria, and this was considered the final dataset for the downstream procedures.

### 2.3. Data Preprocessing

Commonly, after retrieving data from different sources, each entity might have missed information, denoted as blanks, null, or dots. This preprocessing step was employed to eliminate invalid information by removing cases that contained missing information that was necessary for the downstream steps of the ML. Imputation and other replacement procedures were not employed here to prevent the potential addition of bias in the dataset. This preprocessing step did not remove any cases (*n* = 946). By default, the dataset comprised large numeric data values with varying scales. Therefore, we normalized the values accordingly. In this manner, we reduced the impact of outliers, ensured that the machine learning algorithm would operate efficiently, and helped improve the performance of the test and training models. We utilized the min–max scaling range method for normalizing all numerical features between 0 and 1 [20]. The min–max scaling function is available on R packages “caret” version 6.0–92. The normalized datasets were used downstream for classifier model training and testing.

### 2.4. Training and Testing Support Vector Machine (SVM) Classifier

**Figure 1:**
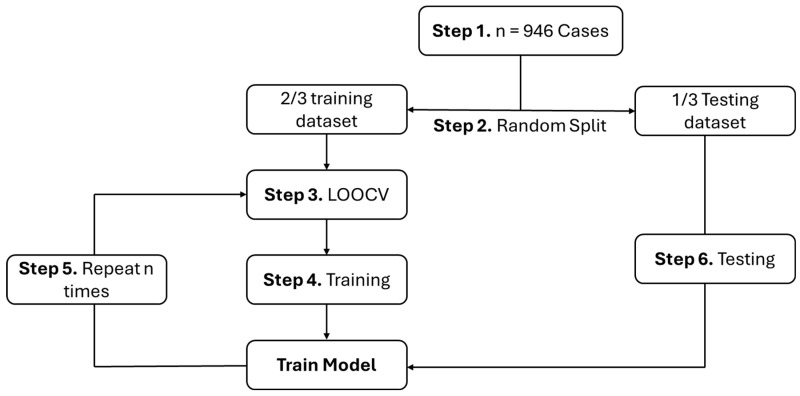
Leave-one-out cross-validation flowchart: Step 1—n = 946 cases (normal and trisomy); Step 2—Split the dataset in testing and training sets; Step 3—A single case is left out to start training; Step 4—Use all cases except the left-out case to train the model; Step 5—Repeat the process n times, with a new single case left each time; Step 6—Test the training model by testing the dataset (this step is repeated 10 times with a new test set selection).

The SVM model has been developed using R packages “e1071” version 1.7-16 [21]. To build the classifier model, the conditions (normal or trisomy) were set as labels, while T4, TSH, age, and gender were considered as variables/features for classifier development. Next, the random splitting of the dataset into a training set (2/3 for model building/training) and a test set (1/3 for model testing) was undertaken. This random split was performed computationally to avoid user selection biases. Additionally, using only one testing set causes the model to rely greatly on the observations of that specific training set. To avoid this issue, the random split was repeated 10 times, and leave-one-out cross-validation (LOOCV) was also performed [10]. The LOOCV procedure works by selecting a single case from the dataset as the testing set and using all other cases as the training set to obtain model performance observations. This process is performed repeatedly with a new case selection for the testing set each time. The final performance observation is the average model performance among the iterations (see Figure 1). This procedure provided much less biased measures in the developed model [10]. This step has been applied to the SVM model with different functions (linear, radial, and polynomial). The average of 10 iterations of model performance for each SVM model type has been tested with a default set of parameters in “e1071” version 1.7-16 (gamma = 1/no. features; cost = 1; epsilon = 0.01; degree = 3 for the polynomial kernel). This default setting was used as a baseline configuration for reasonable starting points for the classification tasks. The performance evaluation has been undertaken according to classifier evaluation metrics. This evaluation aimed to determine the SVM model that could provide a significantly higher performance [22].

### 2.5. Classifier Evaluation Metrics

The performance of each individual classifier (*n* = 10) was evaluated by different performance assessments. (1) The evaluation metrics were the receiver operating characteristic (ROC) curve and area under the curve (AUC) [23] for both the training and the testing sets. This performance assessment provided an overall performance measure across all possible classification thresholds; therefore, the comparison between the classifier functions was facilitated. When the classifier shows an AUC of 1 or closer, this is an indication of successful classifier function, whereas an AUC of 0.50 or less is an indication of an unsuccessful classifier function. (2) Performance values were obtained from 10 trained models by taking average of (A) accuracy, (B) sensitivity, and (C) specificity. Among the trisomy cases, the cases that the classifier correctly categorized as trisomy were grouped as true positive (TP), whereas normal cases correctly categorized as normal were grouped as true negative (TN). Under other conditions, the normal cases incorrectly categorized as trisomy were grouped as false positive (FP), whereas trisomy cases incorrectly classified as normal were grouped as false negative (FN). The TP, TN, FP, and FN were used to calculate the performance values. The classifier with the minimum error rate and maximum accuracy was the most desirable model. The relevant performance values are as follows:

$$Accuracy=\frac{\left(\mathrm{TP}+\mathrm{TN}\right)}{\left(\mathrm{TP}+\mathrm{TN}+\mathrm{FP}+\mathrm{FN}\right)}$$

$$Sensitivity=\frac{\left(\mathrm{TP}\right)}{\left(\mathrm{TP}+\mathrm{FN}\right)}$$

$$Specificity=\frac{\left(\mathrm{TN}\right)}{\left(\mathrm{TN}+\mathrm{FP}\right)}$$

To determine the optimal classification threshold for distinguishing trisomy from normal cases, we applied Youden’s Index to the ROC curves generated from the cross-validation step. Youden’s Index is defined as *J = Sensitivity + Specificity* − 1 and identifies the threshold that maximizes the balance between sensitivity and specificity. This threshold was used as the basis for ChromoCheck’s risk stratification. To enhance clinical interpretability, we defined a three-zone framework—(1) low-risk (green) scores below the threshold; (2) high-risk (red) scores above the threshold; and (3) an intermediate-risk (yellow) zone around the threshold (±0.05)—to account for borderline cases and reduce overconfident classification near decision boundaries

### 2.6. Web Deployment

ChromoCheck v1 was created with the deployment of a high-performance classifier model. In the R studio 2.3.2 environment, a web Shiny app was developed using the following packages: (shiny 1.8.1.1) [24] and (shinythemes 1.2.0) [24]. The combination of these packages provided customized, easy-to-use web applications to host the SVM model. Users can directly provide the input values to predict the ChromoCheck score. The prediction of the deployed model has been optimized by plotting the density of the score among the test datasets. Accordingly, we provided three categories of risk assessment (see Section 3). The ChromoCheck v1 app is freely available at https://nabras-almahrami.shinyapps.io/chromocheck/ (accessed on 30 July 2024). All related files were hosted on the GitHub site https://github.com/nibrasissa (accessed on 30 July 2024).

### 2.7. Case Studies

For the ChromoCheck v1 demonstration, several case studies of suspected trisomy were processed prospectively through a chromosomal study, as well as free T4 and TSH tests. A peripheral blood sample was collected in a lithium heparin tube (2 mL) through standard venipuncture techniques. The chromosome study was performed by culturing 250 µL of peripheral blood lymphocytes in RPMI 1640 medium (Gibco, Thermo Fisher Scientific, Waltham, MA, USA) containing 100 µL phytohemagglutinin (PHA) (Gibco, Thermo Fisher Scientific, Grand Island, NE, USA) to stimulate T-lymphocyte growth and incubating at 37 °C for 72 h. Next, the cells were treated with a colcemid (100 µL) (Gibco, Thermo Fisher Scientific, Grand Island, NE, USA) as a mitotic inhibitor, a hypotonic solution (6 mL of 0.075 M KCl), to swell the cell and separate the chromosomes and the fixative (6 mL of a 1:3 acetic acid–methanol ratio). The fixed cell suspension was dropped into clean microscope slides and air-dried for 1 min. After drying, the slides were stained with a Giemsa stain (G-banding) to identify the dark and light bands, allowing the analysis to proceed. Additionally, free T4 and TSH tests were performed using an automated Atellica CH 930 Analyzer (Siemens Healthineers, Erlangen, Germany). The obtained levels were used for the ChromoCheck score.

## 3. Results

We processed 946 confirmed karyotyping cases (855 normal and 91 trisomy cases) for training and testing through association with the LOOCV principle. The T4, TSH, age, and gender were used as features in this study. Note that we were limited to the available data. Nevertheless, we still effectively obtained the data for machine learning. The generated pre-processed dataset was characterized by a wide range of data values. Consequently, the dataset was normalized. Next, we used the normalized data in the SVM classifier for training and testing. The data was segregated randomly into training and test sets, retaining the distribution of labels (normal vs. trisomy). This process was performed 10 times to ensure that the data splitting process does not generate misleading results. This could happen if a single segregation leads to an overexaggerated strong model performance if, by chance, the split is optimal. The three kernel functions for SVMs (linear, radial, and polynomial kernel) were evaluated through association with the LOOCV principle. The evaluation showed that the SVM classifier using the linear function (training: accuracy, 81%; specificity, 80%; sensitivity, 98%) (training: accuracy, 82%; sensitivity, 97%; specificity, 79%) attained a better performance than other kernel functions with respect to the performance values for training and testing via LOOCV (Table 1).

**Figure 2:**
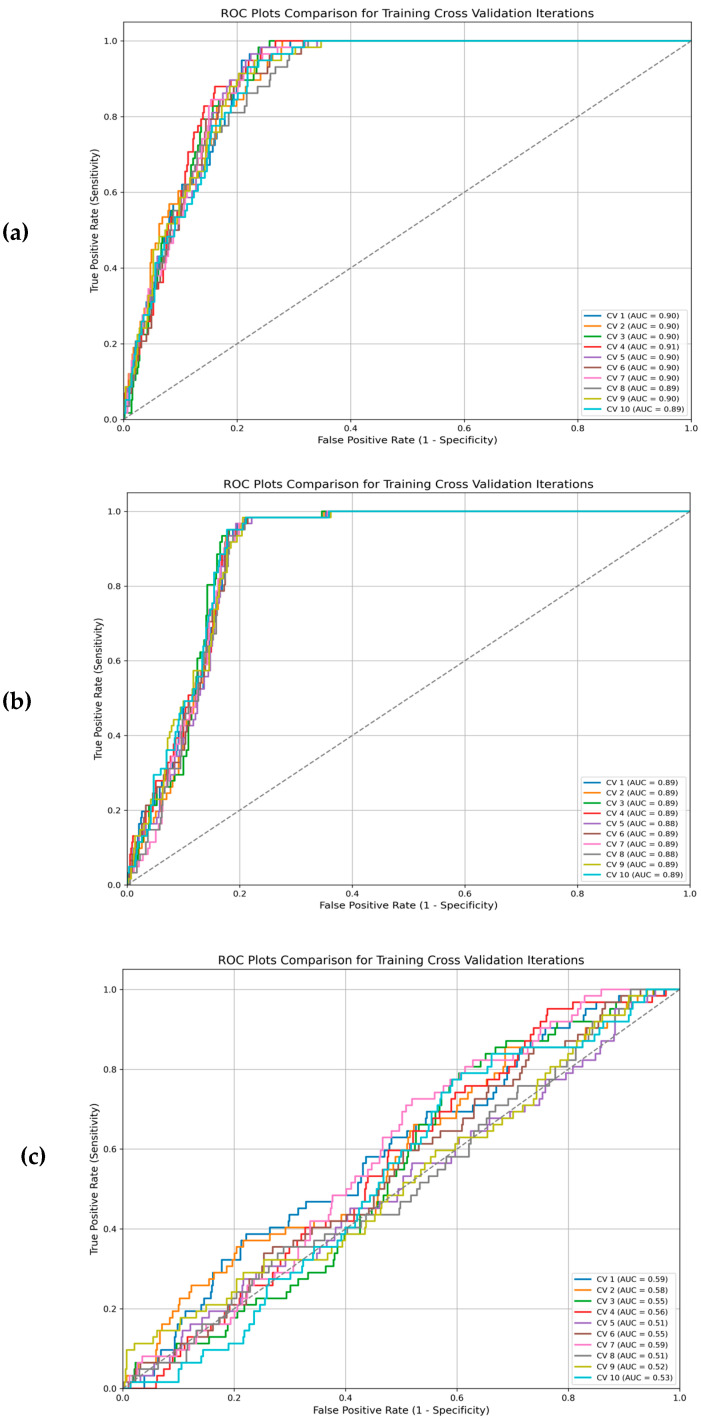
ROC plots comparison for training cross validation iterations with different kernel functions: (**a**) linear kernel function; (**b**) radial kernel function; (**c**) polynomial kernel function.

Furthermore, assessing classifier performance using the area under the ROC curve showed that the linear function (average AUC train: 0.89; average AUC test: 0.90) has a higher performance than the radial (average AUC train: 0.88; average AUC test: 0.89) and polynomial functions (average AUC train: 0.54; average AUC test: 0.87). Results were confirmed via ROC plots analysis for cross-validation of the training set (Figure 2).

**Figure 3:**
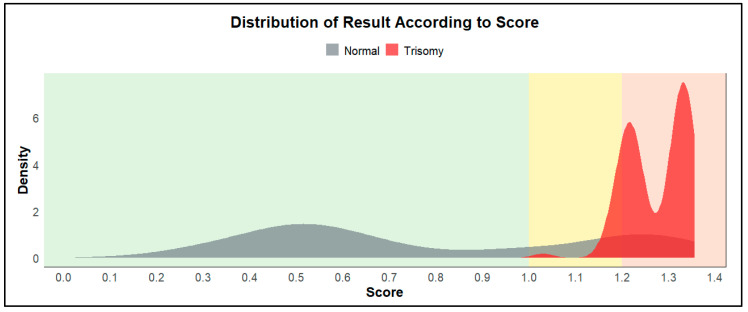
The density plot shows two overlapping distributions of predicted cases. Scores have been grouped into three categories: (1) Green: scores from 0 to 1, indicating a low-risk zone; (2) Yellow: scores from 1 to 1.2, indicating an insignificant zone; (3) Red: scores from 1.2, indicating a high-risk zone.

The optimal threshold derived from the cross-validated training data using Youden’s Index was used to assign risk zones. Figure 3 displays the distribution of ChromoCheck scores across the dataset, with the yellow zone indicating uncertain classifications around the threshold. This stratification revealed a distinct separation between normal and trisomy cases, while also flagging borderline cases for clinical attention. The output scores used in ChromoCheck v1 represent unscaled decision function values from the SVM. These values are based on the distance of each case from the classification hyperplane, and as such, they are not bounded between 0 and 1. Higher scores reflect greater model confidence for trisomy classification, and values may exceed 1 depending on the margin.

**Figure 4:**
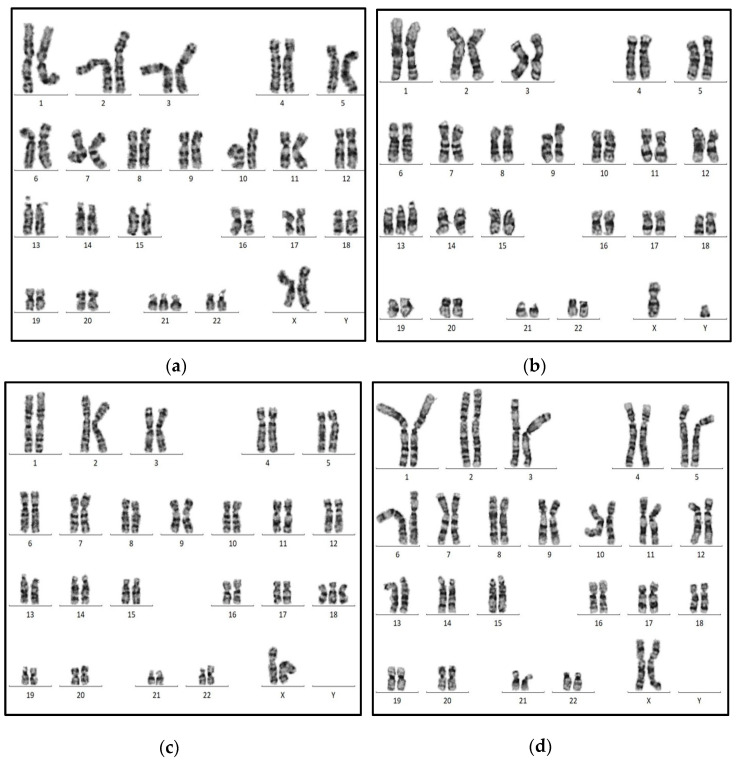
Karyotype analysis of suspected trisomy cases: (**a**) case no.1: Down syndrome (46,XX,+21); (**b**) case no.2: Patau’s syndrome (46,XY,+13); (**c**) case no.3: Edward syndrome (46,XX,+18); (**d**) case no.4: Normal female karyotype (46,XX).

For the ChromoCheck v1 demonstration, four different cases of suspected trisomy conditions have been processed prospectively. In case no.1, a one-day-old female, the clinical examination suggested Down syndrome. The laboratory examinations showed free T4 = 14.4 µIU/mL and TSH = 12.5 µIU/mL. The ChromoCheck score showed 1.29, suggesting a high risk of trisomy, and the karyotype evaluation showed 46,XX,+21 (Figure 4a). Case no.2 is one-day-old male with polydactyly and congenital heart abnormalities. The laboratory examinations showed free T4 = 16.6 µIU/mL and TSH = 0.80 µIU/mL. The ChromoCheck score showed 1.24, suggesting a high risk of trisomy, and the karyotype evaluation showed 46,XY,+13 (Figure 4b). Case no.3 is a two-month-old male with multiple congenital malformations. The lab examinations showed free T4 = 12.7 µIU/mL and TSH = 2.51 µIU/mL. The ChromoCheck score was 1.23, indicating trisomy, and the karyotype evaluation showed 46,XY,+18 (Figure 4c). Case no.4, a 34-days-old female sample, has been processed for chromosomal evaluation due to continuous miscarriage. The lab examinations showed free T4 = 12.1 µIU/mL and TSH = 0.78 µIU/mL. The ChromoCheck score was 0.54, indicating low risk of trisomy condition, and the karyotype evaluation showed normal female 46,XX (Figure 4d).

## 4. Discussion

Genetic conditions such as trisomy cases, which are characterized by the presence of an extra chromosome, can cause several health challenges. The severe forms of trisomy, such as Down syndrome, Edwards syndrome, and Patau syndrome, often require complex healthcare and affect life expectancy. Due to a wide range of clinical features, karyotype and QF-PCR testing have been routinely used in hospital settings to identify numerical abnormalities. Undoubtedly, it is important to develop rapid screening methods to determine trisomy cases. An earlier and more accurate diagnosis assists in the promotion of earlier medical interventions and in the decision-making processes of healthcare professionals and families. According to Fredes D et al. [25], delaying the early intervention program for Down syndrome by more than 6 months is strongly associated with prolonged hospitalization. On the other hand, several studies have provided detailed information on the poor prognosis of patients with Edwards syndrome and Patau syndrome. It has been estimated that the survival rate of both syndromes is less than 14 days, and around 90% of live-born patients do not exceed 1 year [26]. Recently, ML technologies have been employed in various medical applications, including in the diagnosis of genetic disorders, cancer, and psychological disorders. By using large datasets and characterizing hidden patterns, ML can provide a prediction of the diagnosis more quickly and accurately than conventional methods. In this study, a total of 946 cases were newborns screened for trisomy who met the inclusion criteria (855 normal and 91 trisomy cases). The pool of trisomy was a mixture of 81% Down syndrome, 10% Edwards syndrome and 9% Patau syndrome. We processed the datasets to train and test the SVM model. We called the model developed herein ChromoCheck v1. In this version, we assessed the presence of extra chromosomes in suspected cases based on free T4, TSH, gender, and age parameters. ChromoCheck showed a strong performance (average AUC train: 0.89; average AUC test: 0.90) with few required inputs. Although the model showed strong predictive performance, it is important to highlight the class imbalance in the dataset, which represents a common challenge in the classification of rare medical conditions. To reduce potential bias toward the majority class, several strategies have been used as safeguards, including stratified sampling during each split to preserve class ratios across training and test sets, repeated evaluation across 10 randomized training/testing partitions, and using LOOCV with each training set such that all cases, including the minority, contribute to model training. While these strategies reduce the risks of imbalance problems, we recognize that further improvements are possible after the concept is approved. In addition to ROC-AUC, we computed the PR-AUC across all validation folds to more accurately evaluate model performance on the minority class. The aggregated PR-AUC was 0.382, which is substantially higher than the baseline prevalence of trisomy (~9.6%). This suggests that the model captures a signal for trisomy detection beyond random chance, despite the class imbalance. The PR curve is included as Supplementary Figure S1. Additionally, we deployed the trained models into a web tool for simple future utilization. The case studies emphasized the power of utilizing such an approach in augmenting conventional methods of diagnosis and decision-making in a clinical setup.

In previous studies, most of the developed models focused on detecting trisomy 21 based only on prenatal parameters or maternal-associated features. For instance, He F et al. [27] developed a random forest model to predict, prenatally, the risk of Down syndrome pregnancy. The model was trained and tested based on a dataset collected from 86,142 pregnant women, using several features, including free β-human chorionic gonadotrophin (free β-hCG), unconjugated estriol levels (uE3), alpha-fetoprotein concentrations (AFP), maternal age, mother weight, and gestational age. The model achieved an 85% Down syndrome detection rate with 5% false-positives, suggesting an alternative approach for risk assessment. Another study conducted by Zhang L et al. [28] developed a deep learning model based on ultrasonographic images from 800 case–control participants named Trisomy21Net. This model showed significant performance (AUC of 0.98 in the training set and 0.95 in the validation set). Such research endeavors have witnessed the utilization of ML methodologies in predicting trisomy cases. However, we did not find a postnatal ML approach to detecting the pool of different trisomy conditions. Therefore, we propose ChromoCheck v1 to fill this critical gap by providing rapid postnatal screening tools. Moreover, it is also important to pinpoint some limitations of this version. (1) The model was developed using a limited set of features, namely, free T4, TSH, age, and sex, selected for their availability in routine postnatal evaluations. While these variables provide a solid baseline, we recognize that additional features, such as birth weight, Apgar score, craniofacial markers, and other laboratory tests, could enhance ML power. Future work will explore the inclusion of such features to improve model sensitivity, especially for rarer trisomy types. (2) The provided model framework has the potential to be extended to other chromosomal abnormalities. While this study focused specifically on trisomy 13, 18, and 21, similar machine learning methodologies could be adapted to detect conditions such as monosomies. Through these enhancements, ChromoCheck may evolve into a versatile and accessible platform to support early diagnosis and clinical decision-making in genetic medicine in the future.

## 5. Conclusions

In conclusion, we explored using the SVM model with different kernel functions to predict trisomy cases as a postnatal rapid screening model. The ML model was found to be comparable with the conventional karyotype. Finally, we proposed ChromoCheck v1 as a novel web app tool for trisomy detection.

## Figures and Tables

**Table 1:** SVM Performance values of training and testing SVM classifier with different kernel functions.

	Average of Accuracy (%)		Average of Specificity (%)		Average of Sensitivity (%)	
	Train	Test	Train	Test	Train	Test
Linear	81%	82%	79%	80%	97%	98%
Radial	77%	79%	75%	77%	96%	95%
Polynomial	43%	79%	40%	78%	74%	87%

## Data Availability

The datasets generated and analyzed during the current study are not publicly available due to privacy concerns but are available upon reasonable request. For access to the data, please contact the corresponding author, Nabras Al-Mahrami, at nabras.issa@moh.gov.om.

## Supplementary Materials

The following supporting information can be downloaded at https://www.mdpi.com/article/10.3390/genes16060695/s1: Figure S1: PR curve.

## Abbreviations

The following abbreviations are used in this manuscript:

AI	Artificial Intelligence
AFP	Alpha-Fetoprotein
AUC	Area Under the Curve
DS	Down Syndrome
FN	False Negative
FP	False Positive
LOOCV	Leave-One-Out Cross-Validation
ML	Machine Learning
MODY	Maturity Onset Diabetes of the Young
PCR	Polymerase Chain Reaction
QF-PCR	Quantitative Fluorescent Polymerase Chain Reaction
RF	Random Forest
ROC	Receiver Operating Characteristic
STR	Short Tandem Repeat
SVM	Support Vector Machine
T4	Thyroxine Hormone
TN	True Negative
TP	True Positive
TSH	Thyroid-Stimulating Hormone
uE3	Unconjugated Estriol
kNN	k-Nearest Neighbor
k-means	k-Means Clustering
PHA	Phytohemagglutinin
RPMI	Roswell Park Memorial Institute (medium for cell culture)
EIA	Enzyme Immunoassay
CRAN	Comprehensive R Archive Network
CV	Cross-Validation
