# Supplementary figures and images for: ChromoCheck: Predicting Postnatal Chromosomal Trisomy Cases Using a Support Vector Machine Learning Model
Source: Genes (Basel). 2025 Jun 8;16(6):695. doi: 10.3390/genes16060695 (PMC12192111; doi:10.3390/genes16060695)

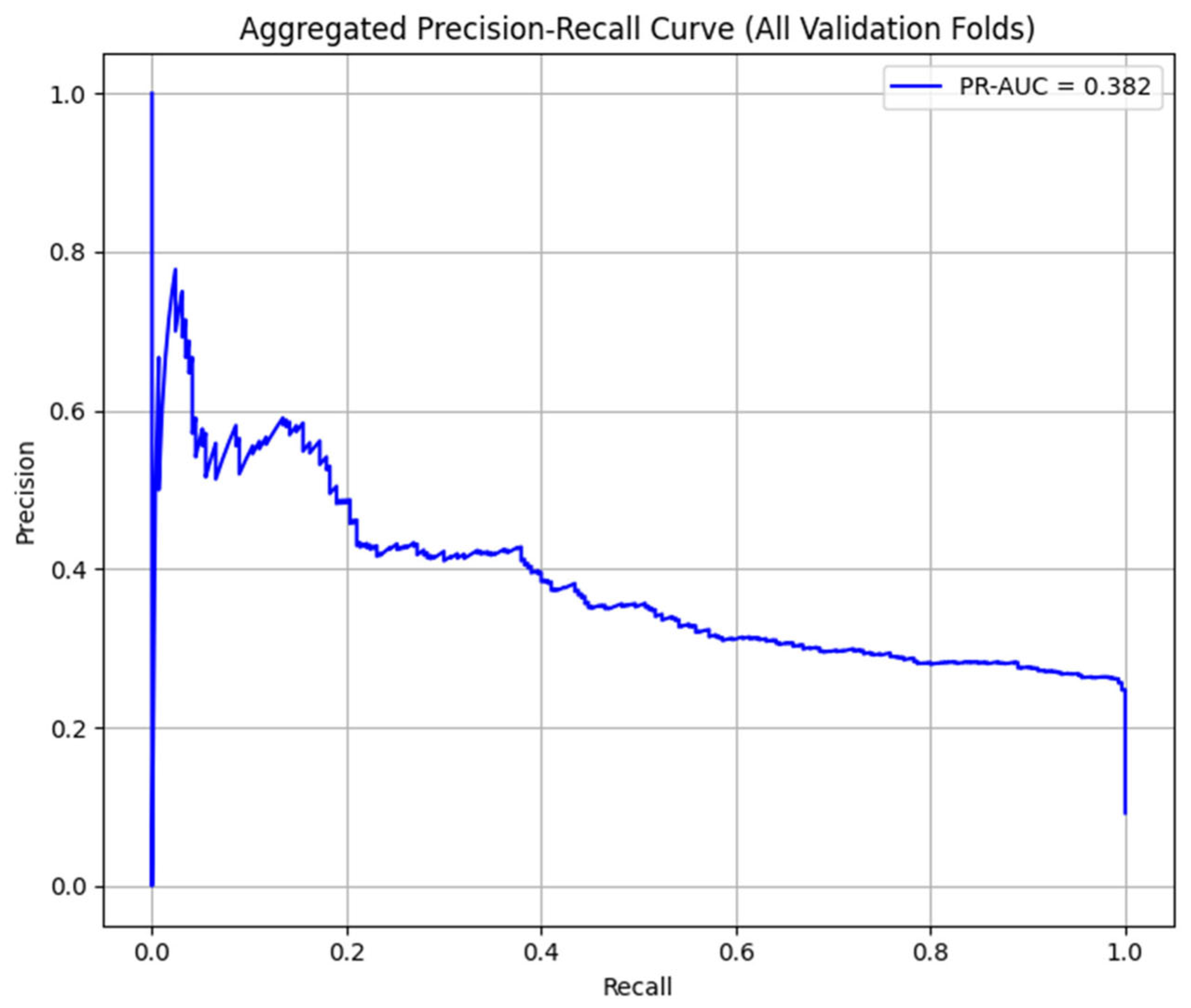

Supplement: Supplementary file 1 [file genes-16-00695-s001.zip › genes-3567722-supplementary.tif]
